# Appraisal of fluorimetric assay of aryl hydrocarbon (benzo(alpha)pyrene) hydroxylase in cultured human lymphocytes.

**DOI:** 10.1038/bjc.1978.207

**Published:** 1978-08

**Authors:** N. M. Trieff, G. C. Forti, V. B. Smart, R. R. KEMPEN, D. J. Kilian


*Br. J. Cancer* (1978) **38**, 335

***Short Communication***

### APPRAISAL OF FLUORIMETRIC ASSAY OF ARYL HYDROCARBON (BENZO(α)PYRENE) HYDROXYLASE IN CULTURED HUMAN LYMPHOCYTES

N. M. TRIEFF†, G. CANTELLI FORTI*, V. B. SMART, R. R. KEMPEN† AND D. J. KILIAN‡

*From the Department of Preventive Medicine and Community Health, University of Texas Medical Branch, Galveston, Texas, *Department of Pharmacology, University of Bologna, Italy, †Department of Pharmacology and Toxicology, University of Texas Medical Branch, Galveston, Texas, ‡Dow Chemical Company, USA, Texas Division, Freeport, Texas, U.S.A.*

Received 24 February 1978 Accepted 5 May 1978

ARYL hydrocarbon hydroxylase (AHH) is an enzyme system found in many tissues and organs within the human body, as well as in various mammalian cells, and which is involved in metabolism of polynuclear aromatic hydrocarbons (PAHs) to more polar derivatives (Heidelberger, 1975). In the case of the metabolism of PAHs such as benzo(α)pyrene (BP) it is thought that one or more of the metabolites is (are) the actual carcinogen(s) (Sims and Grover, 1974; Gelboin *et al.*, 1976; Weinstein *et al.*, 1976; Marquardt, 1977). Using the originally proposed method of Wattenberg *et al.* (1968), Nebert and Gelboin (1968) developed a fluorimetric assay for AHH activity, using liver microsomes for converting BP into 3-hydroxybenzo(α)pyrene (3-BPOH). Several reports have shown that AHH, a mixed-function oxidase, is induced in various tissues by PAHs, drugs, steroids, insecticides and other compounds (Conney, 1967; Nebert and Gelboin, 1968; Gielen and Nebert, 1971, 1972; Lu *et al.*, 1972). Various authors have demonstrated AHH activity in human lymphocytes (Whitlock *et al.*, 1972; Busbee *et al.*, 1972; Kellermann *et al.*, 1973*a*, *b* and *c*; Bast *et al.*, 1976) and have used BP with cultured lymphocytes to determine the AHH activity by fluorimetric measurement of the amount of 3-BPOH formed. The fluorimetric method for AHH activity in human lymphocytes has created much interest as a relatively simple screening test for susceptibility to bronchogenic cancer (Kellermann *et al.*, 1973*b*, *c* and *d*) as caused by either cigarette smoking or various other chemical agents (Alfred and Bowens, 1975). Nevertheless, the test does not appear very reproducible either in a single laboratory or between laboratories (Paigen *et al.*, 1977; Cantelli Forti *et al.*, 1977). Hence, many variants of the original fluorimetric method of Wattenberg *et al.* (1968) and Nebert and Gelboin (1968) have been published by different workers in an attempt to improve its reproducibility and accuracy (Whitlock *et al.*, 1972; Busbee *et al.*, 1972; Kellermann *et al.*, 1973*a*, *b* and *c*; Dehnen *et al.*, 1973; Gurtoo *et al.*, 1975; Cantrell *et al.*, 1976; Bast *et al.*, 1976; Paigen *et al.*, 1977). It is the purpose of this communication to examine some of the problems inherent in the fluorimetric assay of AHH in human lymphocytes.

Six simulated experiments were performed in test tubes, precisely according

Correspondence: Prof. Norman M. Trieff, Environmental Toxicology Department of Preventive Medicine and Community Health, University of Texas Medical Branch, Galveston, Texas 77550, USA.

to the procedure developed by Kellermann *et al.* (1973*a*, and *b*) for determination of AHH activity by the fluorimetric procedure, except for the fact that lymphocytes were omitted from the test tubes. Each test tube contained 0·9 ml of a buffer mixture (TMS, see Busbee *et al.*, 1972) consisting of TRIS (final concentration 50 mM), $MgCl_2$ (final concentration of 3 mM) and sucrose (final concentration 200 mM). In addition there was added 0·70 mg NADH+0·70 mg NADPH in a volume of 0·10 ml of TMS. The freshly prepared mixture was incubated immediately after the addition of unlabelled BP (25 μg) mixed with $25\times 10^{-3}$ μCi of labelled BP in 50 μl of acetone.

The unlabelled BP (98% pure, m.p. 175–177°C, Aldrich Chemical Co.) and the generally tritiated benzo(α)pyrene ([G-$^3$H] BP) (Amersham/Searle Co; sp. act. 5 Ci/mmol; 20 mCi/mg; radioactive concentration, 5 mCi/ml in benzene; BP concentration, 0·250 mg/ml) were purified by thin-layer chromatography 2-dimensionally on silica gel G (Merck pre-coated plates) using first, benzene and then 1:15 (v/v) benzene: hexane. The major, 0·90 $R_f$, fraction was retained and an acetone stock solution was prepared for the experiments.

Incubation of the test tubes was carried out in a shaking bath at 37±0·05°C for 30 min. At the end of this time, 4 ml of 25% acetone in hexane was added. The tubes were mixed on a vortex for 1 min and the phases separated by centrifugation at 400 *g* for 3 min.

The buffered aqueous phase (A) was recovered and transferred directly into a liquid-scintillation vial containing 10 ml of PCS™ cocktail (Phase Combining System^TRADEMARK Amersham/Searle Co.). The organic phase was vortexed in another test tube with 1 ml of 1N NaOH. After additional mixing for 1 min and centrifugation, the phases were separated. The organic phase (B) and the NaOH phase (C) were each transferred into 10 ml of PCS™ cocktail in liquid-scintillation vials.

Standards of buffer mixture, hexane: acetone (1:3 v/v) mixture, 1N NaOH and BP solution (unlabelled) were added separately to 10 ml of PCS™ cocktail, to obtain the quenching calibration curves. A Beckman model LS-100 was used for radioactive measurements.

All experiments were made using yellow light, because of the observed and widely known photo-decomposition of BP and its metabolites. Fluorescence spectra were obtained on a Perkin-Elmer Model MPF-2A, using an excitation wavelength of 396 nm.

TABLE I.—*Percent recovered radioactivity in various phases using [G-$^3$H]BP in Kellermann* et al. (1973a, b) *procedure for fluorimetric assay*

| | Mean ±s.e. n=6 | Confidence limits ($P<0·05$) |
|---|---|---|
| % of total activity found in the TMS buffer phase (A) | 0·29±0·06 | 0·12<br>0·45 |
| % of total activity found in the organic phase (B) | 86·88±1·85 | 82·11<br>91·65 |
| % of total activity found in the 1N NaOH phase (C) | 11·72±0·82 | 9·61<br>13·82 |
| % Total recovered activity | 98·89±2·42 | 92·72<br>105·06 |

Table I shows the percentage of total radioactivity found in the various phases, using the procedure of Kellermann *et al.* (1973*a*, and *b*) in the absence of human lymphocytes but with [G-$^3$H]BP. The most significant fact is that 11·72% of the total radioactivity ends up in the NaOH phase (C), while the method was developed to ensure that no BP, only 3-BPOH, was being extracted by the NaOH. It should be noted that the total recovery of radioactivity is 98·89%, suggesting minimal error due to losses.

On the basis of the data in Table I, fluorimetric scans were made on various concentrations of 3-BPOH in 1N NaOH. For each concentration, a scan was made in the absence and presence of 6 μl of BP solution (0·50 μg/μl) added to each solution after measuring the 3-BPOH alone. This amount of added BP represents 11% of the total amount of BP added to the

TABLE II.—*Fluorimetric measurements on 3-BPOH and (3-BPOH+BP) for different concentrations of 3-BPOH**

| | 3-BPOH concentration (M) | Fluorescence: 3-BPOH only | Fluorescence: 3-BPOH+6 μl of 0·5 μg/μl BP solution | Ratio |
|---|---|---|---|---|
| Slits: emission 5 excitation 5 | $10^{-5}$ | 240 | 310 | 1·29 |
| | $10^{-6}$ | 20 | 128 | 6·40 |
| | $10^{-7}$ | 4 | 70 | 12·50 |
| | $10^{-8}$ | 2 | 55 | 27·50 |
| | $10^{-9}$ | 1 | 35 | 35·00 |
| Slits: emission 10, excitation 10 | $10^{-9}$ | 95 | 195 | 2·05 |
| | $10^{-10}$ | 43 | 168 | 3·91 |
| | $10^{-11}$ | 28 | 120 | 4·29 |
| | $10^{-12}$ | 14 | 84 | 6·00 |

* Perkin-Elmer fluorescence spectrophotometer Model MPF-2A; excitation 396 nm; emission 522 nm; sensitivity 4. Values were obtained from recorded scans using a fixed excitation at 396 nm.

† Arbitarary units.

lymphocytes in the modified fluorimetric procedure of Kellermann *et al.* (1973*a*, and *b*) close to that percentage found in Phase C (Table I). The results of the study are summarized in Table II. It is clear that at high concentrations of 3-BPOH ($<10^{-5}$M) there is not much difference between the fluorescence values. However, as the concentrations of 3-BPOH decrease, a substantial positive error occurs, as shown by the increasing ratio. Data in Table I also show the effect of slit width. At $10^{-9}$M 3-BPOH, slits of 5 give a ratio of 35, while slits of 10 lead to a ratio of 2. Depending on the slit width and concentration, the ratio of fluorescence intensity of the solution with BP to that without BP may be as large as 35:1 or as small as 1·3:1. Clearly, the larger the slit widths for excitation and emission, the smaller the ratio, although a larger slit width decreases the slope of the log fluorescence *vs*-log concentration curve (not shown).

The implications of these results are: (1) that ~ 11·7% of the BP is extracted by the NaOH in the fluorimetric method, (2) that its fluorescence spectrum has one peak with a wavelength maximum at 522 nm, the same as for 3-BPOH; hence there is a substantial interference of the BP in the measurement of the fluorescence intensity of 3-BPOH, (3) as the concentration of 3-PBOH decreases and the slit width decreases, this effect becomes more substantial.

Inducibility as noted by Kellermann *et al.* (1973*b* and *c*) is measured by comparing the fluorescence intensity at 522 nm (excitation at 396 nm) with 3-methylcholanthrene (3-MC) incubation to that without 3-MC addition. This ratio ×100 is the percent inducibility. Assuming no actual inducibility, if the amount of BP extracted from the split lymphocyte sample (induced and uninduced) varies, there will be either an apparent positive induction or negative induction, depending upon which half extracts the greater amount of BP. Thus, the apparent inducibility as measured by fluorescence may be a complete artifact, due to unequal BP contamination in the "induced" and "uninduced" samples.

This point is emphasized by our results using the conventional fluorimetric procedure modified by Kellerman *et al.* (1973*a* and *b*) for AHH assay on human lymphocytes. Out of 68 samples, 24 showed a negative induction. The observed negative induction may be in part due to an artifact in the procedure, as noted, resulting from fluorimetric interference of BP. It also may be a true effect resulting from the interaction of various individual genetic and environmental factors (Cantelli Forti

*et al.*, 1977). Also, although we have not yet quantitated this, other metabolites present may interfere, as shown by Holder *et al.* (1975). Data on the presence of other metabolites as detected by radioisotopic assay developed by us will be presented elsewhere (Cantelli Forti *et al.*, 1977).

In summary, the fluorimetric procedure such as that of Kellermann *et al.* (1973*a* and *b*) for AHH inducibility in human lymphocytes has a number of inherent problems. These are: (1) the extraction of BP along with 3-BPOH, and probably other metabolites, and their fluorimetric interference, (2) the photochemical instability of the BP, 3-BPOH and other metabolites which may lead to additional errors if proper precautions are not taken. This paper emphasizes the first point.

We are grateful for the generous support of this work by Dow Chemical Co., U.S.A. We also wish to acknowledge the kind gift of 3-hydroxybenzo[*a*]-pyrene by Dr Harry V. Gelboin, N.C.I., Bethesda, MD, USA.

REFERENCES

ALFRED, L. J. & BOWENS, M. P. (1975) Human lymphocyte AHH activity testing; possible cancer risk predictor. *UCLA Cancer Center Bull.*, **2**, 8.

BAST, R. C., JR, OKUDA, T., PLOTKIN, E., TARONE, R., RAPP, H. J. & GELBOIN, H. V. (1976) Development of an assay for aryl hydrocarbon benzo(a) pyrene hydroxylase in human peripheral blood monocytes. *Cancer Res.*, **36**, 1967.

BUSBEE, D. L., SHAW, C. R. & CANTRELL, E. T. (1972) Aryl hydrocarbon hydroxylase induction in human leukocytes. *Science*, **178**, 315.

CANTELLI FORTI, G., TRIEFF, N. M., BUNCE III, H. & KILIAN, J. (1977) TLC radioisotopic assay of aryl hydrocarbon hydroxylase (AHH) activity in human lymphocytes. Presented at the Venice Joint Meeting of German and Italian Pharmacologists (Abstr.).

CANTRELL, E., ABREU, M. & BUSBEE, D. (1976) A simple assay of aryl hydrocarbon hydroxylase in cultured human lymphocytes. *Biochem. Biophys. Res. Comm.*, **70**, 474.

CONNEY, A. H. (1967) Pharmacological implications of microsomal enzyme induction. *Pharmacol. Rev.*, **19**, 317.

DEHNEN, W., TOMINGAS, R. & ROSS, J. (1973) A modified method for the assay of benzo(a)pyrene hydroxylase. *Anal. Biochem.*, **53**, 373.

GELBOIN, H. V., SELKIRK, J. K., YANG, S. K., WIEBEL, F. J. & NEMOTO, N. (1976) Benzo(a) pyrene metabolism by mixed-function oxygenases, hydratases, and glutathione S-transferases: analysis by high pressure liquid chromatography. In *Glutathione: Metabolism and Function.* Ed. I. M. Arias and W. B. Jakoby. New York: Raven Press.

GIELEN, J. E. & NEBERT, D. W. (1971) Microsomal hydroxylase induction in liver cell culture by phenobarbital, polycyclic hydrocarbons and p,p'-DDT. *Science*, **172**, 167.

GIELEN, J. E. & NEBERT, D. W. (1972) Aryl hydrocarbon hydroxylase induction in mammalian liver cell culture: III. Effect of various sera, hormone, biogenic amines and other endogenous compounds on the enzyme activity. *J. Biol. Chem.*, **247**, 7591.

GURTOO, H. L., BEJBA, N. & MINOWADA, J. (1975) Properties, inducibility, and improved method of analysis of aryl hydrocarbon hydroxylase in cultured human lymphocytes. *Cancer Res.*, **35**, 1235.

HEIDELBERGER, C. (1975) Chemical carcinogenesis. *Ann. Rev. Biochem.*, **44**, 79.

HOLDER, G., YAGI, H., LEVIN, W., LU, A. Y. H. & JERINA, D. M. (1975) Metabolism of benzo(a) pyrene. III. An evaluation of the fluorescence assay. *Biochem. Biophys. Res. Comm.*, **65**, 1363

KELLERMANN, G., CANTRELL, E. & SHAW, C. R. (1973*a*) Variations in extent of aryl hydrocarbon hydroxylase induction in cultured human lymphocytes. *Cancer Res.*, **33**, 1654.

KELLERMANN, G., LUYTEN-KELLERMANN, M. & SHAW, C. R. (1973*b*) Genetic variations of aryl hydrocarbon hydroxylase in human lymphocytes. *Am. J Human Genet.*, **25**, 327

KELLERMANN, G, LUYTEN-KELLERMANN, M. & SHAW, C. R. (1973*c*) Metabolism of polycyclic aromatic hydrocarbons in cultured human leukocytes under genetic control. *Humangenetik*, **20**, 257.

KELLERMANN, G., SHAW, C. R. & LUYTEN-KELLERMANN, M. (1973*d*) Aryl hydrocarbon hydroxylase inducibility and bronchogenic carcinoma *New Engl. J. Med.*, **289**, 934.

LU, A. Y. H., SOMOGYI, A., WEST, S., KUNTZMAN, R. & CONNEY, A. H. (1972) Prepnenolone-16a-carbonitrile: a New type of inducer of drug-metabolizing enzyme. *Arch. Biochem. Biophys.*, **152**, 457.

MARQUARDT, H. (1977) Microsomal metabolism of chemical carcinogens in animals and in man. In *Air Pollution and Cancer in Man.* Ed. U. Mohr, D. Schmall and L. Tomatis. Lyon: IARC Scient. Pub. p. 309.

NEBERT, D. W. & GELBOIN, H. V. (1968) Substrate-inducible microsomal aryl hydroxylase in mammalian cell culture II. *J. Biol. Chem.*, **243**, 6242.

PAIGEN, B., GURTOO, H. L., MINOWADA, J., HOUTEN, L., VINCENT, R., PAIGEN, K., PARKER, N. B., WARD, E. & HAYNER, N. T. (1977) Questionable relations of aryl hydrocarbon hydroxylase to lung-cancer risk. *New Engl. J. Med.*, **297**, 346.

SIMS, P. & GROVER, P. L. (1974) Epoxides in polycyclic aromatic hydrocarbon metabolism and carcinogenesis. *Adv. Cancer Res.*, **20**, 165.

WATTENBERG, L. W., LEONG, J. L. & GALBRAITH, A. R. (1968) Induction of increased benzopyrene hydroxylase activity in pulmonary tissue *in vitro. Proc. Soc. Exp. Biol. Med.*, **127**, 467.

WEINSTEIN, I. B., JEFFREY, A. A., JENNETTE, K. W., BLOBSTEIN, S. H., HARVEY, R. G., HARRIS, C., AUTRUP, H., KASAI, H. & NAKANISHI, K. (1976) Benzo(a)pyrene diol epoxides as intermediates in nucleic acid binding *in vitro* and *in vivo. Science*, **193**, 592.

WHITLOCK, J. P., COOPER, H. L. & GELBOIN, H. V. (1972) Aryl hydrocarbon (benzo(a)pyrene) hydroxylase is stimulated in human lymphocytes by mitogens and benzo(a)anthracene. *Science*, **177**, 618.


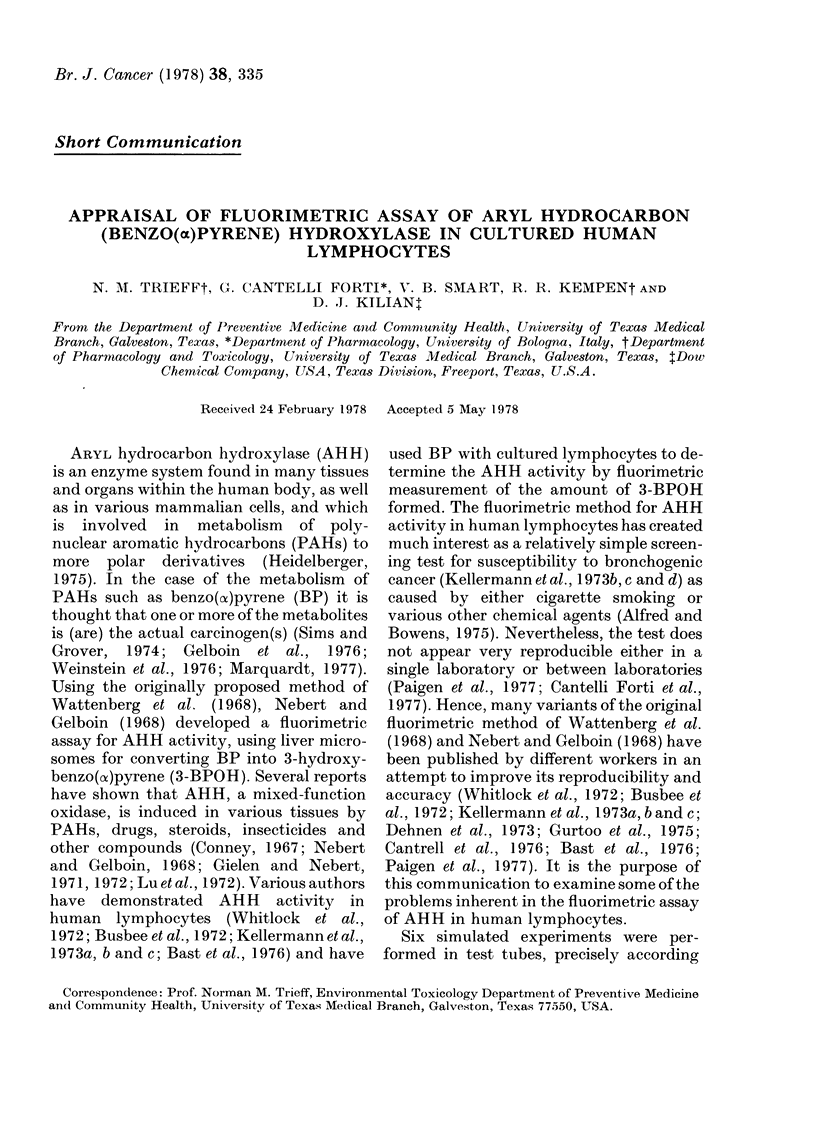

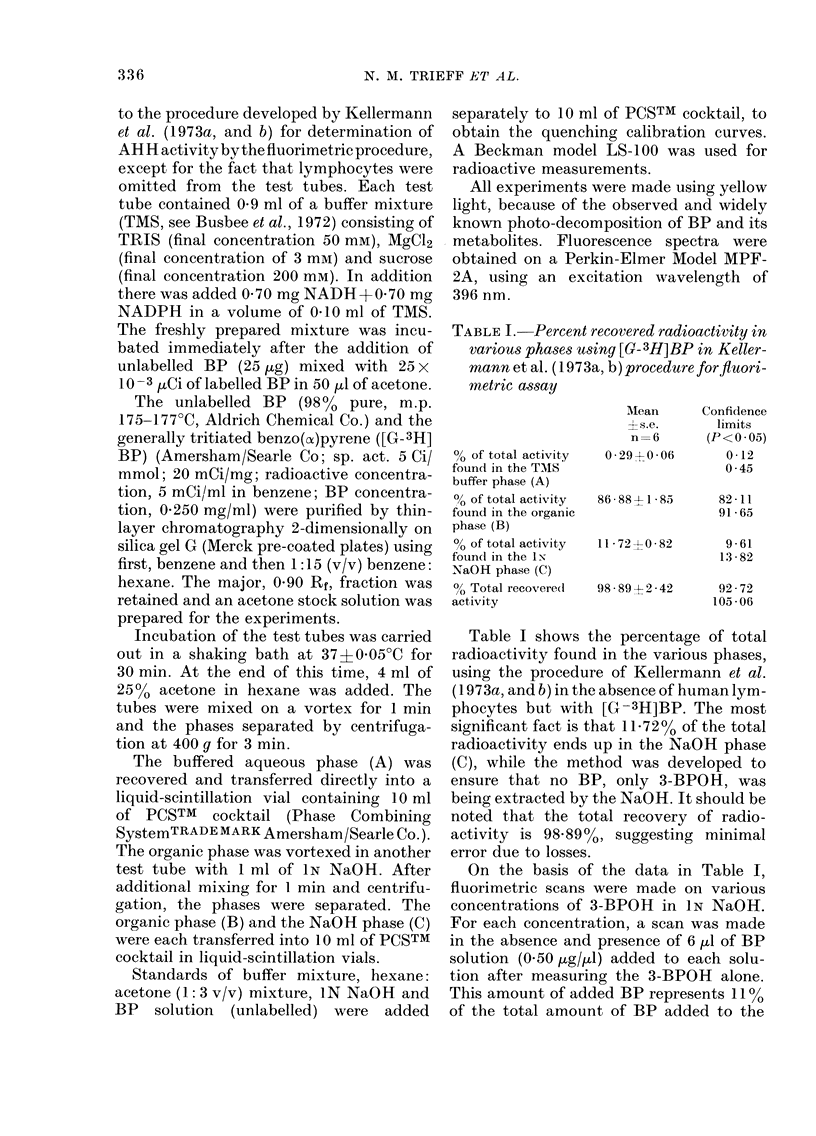

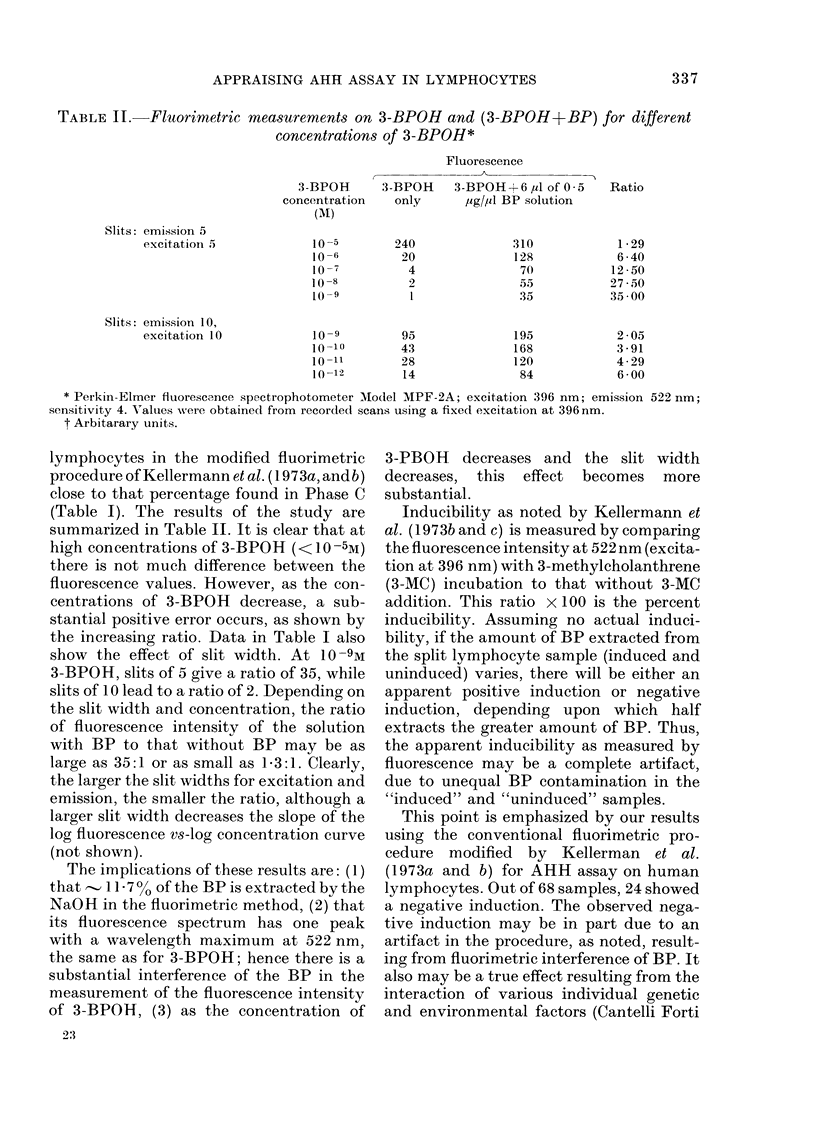

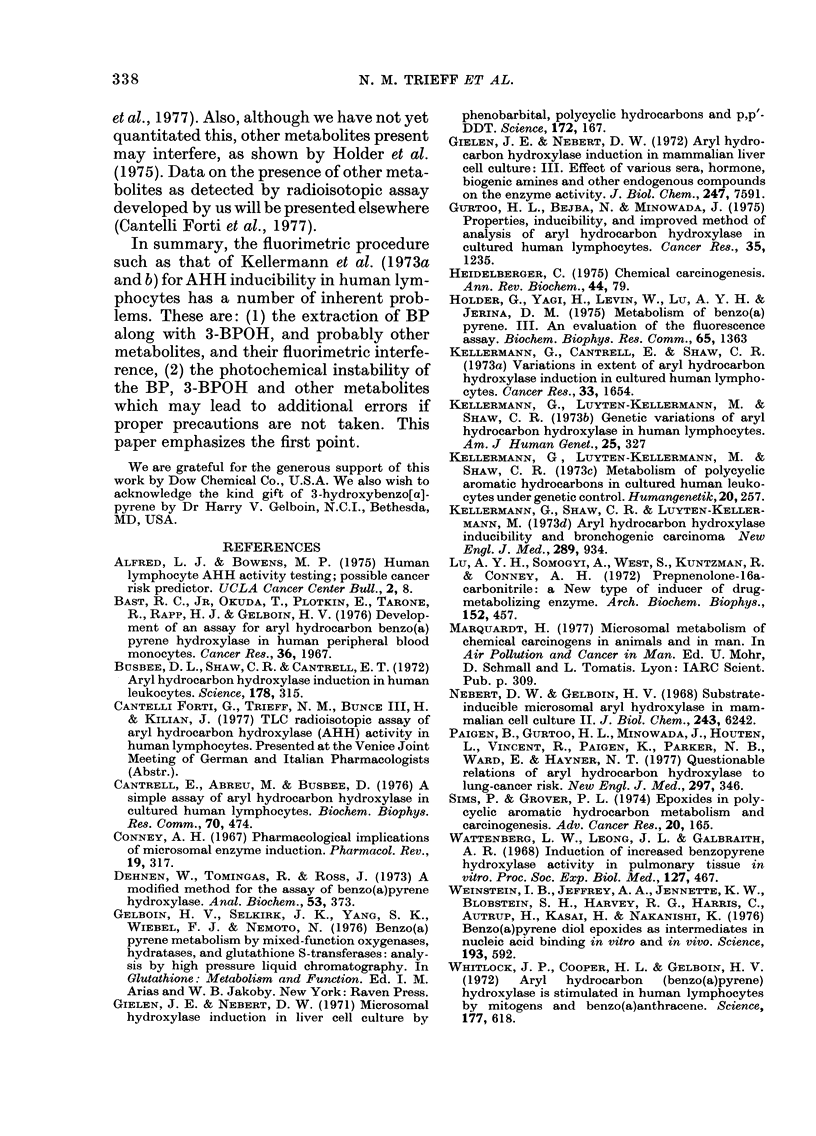


## References

[OCR_00412] Bast R. C., Okuda T., Plotkin E., Tarone R., Rapp H. J., Gelboin H. V. (1976). Development of an assay for aryl hydrocarbon (benzo(a)pyrene) hydroxylase in human peripheral blood monocytes.. Cancer Res.

[OCR_00419] Busbee D. L., Shaw C. R., Cantrell E. T. (1972). Aryl hydrocarbon hydroxylase induction in human leukocytes.. Science.

[OCR_00432] Cantrell E., Abreu M., Busbee D. (1976). A simple assay of aryl hydrocarbon hydroxylase in cultured human lymphocytes.. Biochem Biophys Res Commun.

[OCR_00438] Conney A. H. (1967). Pharmacological implications of microsomal enzyme induction.. Pharmacol Rev.

[OCR_00443] Dehnen W., Tomingas R., Roos J. (1973). A modified method for the assay of benzo(a)pyrene hydroxylase.. Anal Biochem.

[OCR_00463] Gielen J. E., Nebert D. W. (1972). Aryl hydrocarbon hydroxylase induction in mammalian liver cell culture. 3. Effects of various sera, hormones, biogenic amines, and other endogenous compounds on the enzyme activity.. J Biol Chem.

[OCR_00456] Gielen J. E., Nebert D. W. (1971). Microsomal hydroxylase induction in liver cell culture by phenobarbital, polycyclic hydrocarbons, and p,p'-DDT.. Science.

[OCR_00469] Gurtoo H. L., Bejba N., Minowada J. (1975). Properties, inducibility, and an improved method of analysis of aryl hydrocarbon hydroxylase in cultured human lymphocytes.. Cancer Res.

[OCR_00476] Heidelberger C. (1975). Chemical carcinogenesis.. Annu Rev Biochem.

[OCR_00480] Holder G., Yagi H., Levin W., Lu A. Y., Jerina D. M. (1975). Metabolism of benzo[a]pyrene. III. An evaluation of the fluorescence assay.. Biochem Biophys Res Commun.

[OCR_00486] Kellermann G., Cantrell E., Shaw C. R. (1973). Variations in extent of aryl hydrocarbon hydroxylase induction in cultured human lymphocytes.. Cancer Res.

[OCR_00492] Kellermann G., Luyten-Kellermann M., Shaw C. R. (1973). Genetic variation of aryl hydrocarbon hydroxylase in human lymphocytes.. Am J Hum Genet.

[OCR_00498] Kellermann G., Luyten-Kellermann M., Shaw C. R. (1973). Metabolism of polycyclic aromatic hydrocarbons in cultured human leukocytes under genetic control.. Humangenetik.

[OCR_00509] Lu A. Y., Somogyi A., West S., Kuntzman R., Conney A. H. (1972). Pregnenolone-16 -carbonitrile: a new type of inducer of drug-metabolizing enzymes.. Arch Biochem Biophys.

[OCR_00523] Nebert D. W., Gelboin H. V. (1968). Substrate-inducible microsomal aryl hydroxylase in mammalian cell culture. I. Assay and properties of induced enzyme.. J Biol Chem.

[OCR_00528] Paigen B., Gurtoo H. L., Minowada J., Houten L., Vincent R., Paigen K., Parker N. B., Ward E., Hayner N. T. (1977). Questionable relation of aryl hydrocarbon hydroxylase to lung-cancer risk.. N Engl J Med.

[OCR_00535] Sims P., Grover P. L. (1974). Epoxides in polycyclic aromatic hydrocarbon metabolism and carcinogenesis.. Adv Cancer Res.

[OCR_00540] Wattenberg L. W., Leong J. L., Galbraith A. R. (1968). Induction of increased benzpyrene hydroxylase activity in pulmonary tissue in vitro.. Proc Soc Exp Biol Med.

[OCR_00546] Weinstein I. B., Jeffrey A. M., Jennette K. W., Blobstein S. H., Harvey R. G., Harris C., Autrup H., Kasai H., Nakanishi K. (1976). Benzo(a)pyrene diol epoxides as intermediates in nucleic acid binding in vitro and in vivo.. Science.

[OCR_00554] Whitlock J. P., Cooper H. L., Gelboin V. H. (1972). Aryl hydrocarbon (benzopyrene) hydroxylase is stimulated in human lymphocytes by mitogens and benz(a)anthracene.. Science.

